# Correction: A coupling process of electrodialysis with oxime hydrolysis reaction for preparation of hydroxylamine sulfate

**DOI:** 10.1039/d1ra90126e

**Published:** 2021-07-05

**Authors:** Fenggang Guan, Yanyan Chen, Yuying Zhang, Rujun Yu, Li Xue

**Affiliations:** School of Chemistry and Chemical Engineering, Shandong University of Technology Zibo 255049 PR China zbyurj@163.com

## Abstract

Correction for ‘A coupling process of electrodialysis with oxime hydrolysis reaction for preparation of hydroxylamine sulfate’ by Fenggang Guan *et al.*, *RSC Adv.*, 2021, **11**, 19238–19247, DOI: 10.1039/D1RA02766B.

The authors regret the omission of one of the authors, Li Xue, from the original manuscript. The corrected author list is as shown above.

The Royal Society of Chemistry apologises for these errors and any consequent inconvenience to authors and readers.

## Supplementary Material

